# Upregulation of ERK1/2-eNOS via AT2 Receptors Decreases the Contractile Response to Angiotensin II in Resistance Mesenteric Arteries from Obese Rats

**DOI:** 10.1371/journal.pone.0106029

**Published:** 2014-08-29

**Authors:** Graziela N. Hagihara, Nubia S. Lobato, Fernando P. Filgueira, Eliana H. Akamine, Danielle S. Aragão, Dulce E. Casarini, Maria Helena C. Carvalho, Zuleica B. Fortes

**Affiliations:** 1 Department of Pharmacology, Institute of Biomedical Sciences, University of Sao Paulo, Sao Paulo, Brazil; 2 Department of Biological Sciences, Division of Cardiovascular Physiology, Federal University of Goias, Jatai, Brazil; 3 Department of Medicine, Division of Nephrology, Escola Paulista de Medicina, Federal University of Sao Paulo, Sao Paulo, Brazil; Max-Delbrück Center for Molecular Medicine (MDC), Germany

## Abstract

It has been clearly established that mitogen-activated protein kinases (MAPKS) are important mediators of angiotensin II (Ang II) signaling via AT1 receptors in the vasculature. However, evidence for a role of these kinases in changes of Ang II-induced vasoconstriction in obesity is still lacking. Here we sought to determine whether vascular MAPKs are differentially activated by Ang II in obese animals. The role of AT2 receptors was also evaluated. Male monosodium glutamate-induced obese (obese) and non-obese Wistar rats (control) were used. The circulating concentrations of Ang I and Ang II, determined by HPLC, were increased in obese rats. Ang II-induced isometric contraction was decreased in endothelium-intact resistance mesenteric arteries from obese compared with control rats and exhibited a retarded AT1 receptor antagonist response. Blocking of AT2 receptors and inhibition of either endothelial nitric oxide synthase (eNOS) or extracellular signal-regulated protein kinases 1 and 2 (ERK1/2) restored Ang II-induced contraction in obese rats. Western blot analysis revealed increased protein expression of AT2 receptors in arteries from obese rats. Basal and Ang II-induced ERK1/2 phosphorylation was also increased in obese rats. Blockade of either AT1 or AT2 receptors corrected the increased ERK1/2 phosphorylation in arteries from obese rats to levels observed in control preparations. Phosphorylation of eNOS was increased in obese rats. Incubation with the ERK1/2 inhibitor before Ang II stimulation did not affect eNOS phosphorylation in control rats; however, it corrected the increased phosphorylation of eNOS in obese rats. These results clearly demonstrate that enhanced AT2 receptor and ERK1/2-induced, NO-mediated vasodilation reduces Ang II-induced contraction in an endothelium-dependent manner in obese rats.

## Introduction

Angiotensin II (Ang II), the effector peptide of the renin-angiotensin system (RAS), is a vasoactive peptide that exerts a variety of vascular actions through activation of at least two different types of G protein–coupled receptors, the type 1 receptor (AT1) and the type 2 receptor (AT2) [1]–[3]. Binding of Ang II to the AT1 receptor activates a myriad of signaling pathways, among them the mitogen-activated protein kinases (MAPKS), a family of serine/threonine kinases which are classically associated with vascular smooth muscle cell contraction, migration, adhesion, collagen deposition, cell growth, differentiation, and survival. Of the main MAPKs, extracellular signal-regulated kinases (ERK1/2), p38 MAPK, and stress-activated protein kinase/c-Jun N-terminal kinases (SAPK/JNK) are the best characterized [4]–[9].

Although Ang II signaling via AT1 receptor has been extensively characterized, Ang II signaling via AT2 receptors is still not completely understood. In small resistance vessels, activation of AT1 receptors promotes vasoconstriction and smooth muscle proliferation [10], whereas AT2 receptor stimulation activates an autacoids vasodilator cascade composed of bradykinin (BK), nitric oxide (NO), and guanosine cyclic 3, 5 -monophosphate (cGMP) that mediates vasodilation, counteracting AT1 receptor-induced contraction and providing a protective role [11], [12]. Indeed, AT2 receptor knockout mice have higher blood pressure and an exaggerated response to Ang II infusion on blood pressure [13]. Furthermore, AT2 receptor is upregulated in certain pathological conditions such as hypertension, vascular injury, and inflammation [14], [15]. The significance of AT2 receptor in the establishment of vascular dysfunction in obesity, however, is not defined.

Recent studies have demonstrated that Ang II plays an important role in obesity by promoting changes in energetic homeostasis and vascular function [16]–[17]. Increased activation of MAPKs has also been described to be involved is changes of the energy metabolism in obesity [18]–[20]. Moreover, it is now clearly established that MAPKs are important mediators of Ang II effects in the vasculature, including vascular smooth muscle cells differentiation, proliferation, migration, and fibrosis [21], [22]. However, evidence for a direct role of these kinases in changes of the vascular reactivity to Ang II in obesity is still lacking. Here we sought to determine whether MAPKs activation, in particular ERK1/2, p38 MAPK, and JNK, are differentially regulated by Ang II in vessels from obese animals. The role of AT2 receptors was also evaluated. To address these issues, we used resistance mesenteric arteries from male monosodium glutamate (MSG)-induced obese rat, a model of obesity with insulin resistance and dyslipidemia that may occur without the presence of type II diabetes or hypertension depending on the age at which the animals are studied [23], making them a relevant model to investigate the vascular dysfunction associated with obesity. Our results showed that activation SAPK/JNK and p38MAPK pathways contribute to the maintenance of vasoconstriction to Ang II via AT1 receptors while activation of ERK1/2-eNOS pathway via AT2 receptors in the endothelium contributes to counteracting contraction and decrease the response to Ang II in this experimental model of obesity.

## Methods

### Animals, Induction and Characterization of Obesity

All animal procedures were approved by the Ethical Committee for Animal Research of the Institute of Biomedical Sciences, University of Sao Paulo, conformed to the Guide for the Care and Use of Laboratory Animals published by the US National Institutes of Health (NIH Publication No. 85–23, revised 1996). Male Wistar rats received subcutaneous injections of MSG (4.0 g/kg) dissolved in 0.9% NaCl (obese rats) or an equivalent volume of vehicle (control rats), from the second to sixth day after birth. The breeding conditions were followed as previously described [23]. All experimental groups were studied at 16 weeks of age. The water and food consumption was determined by placing the rats in metabolic cages. Rats were acclimated for 72 h and data were collected over the next 24 h.

On the day of the experiment, after food deprivation (5 h), obese and control rats were weighed, and blood samples were taken from the descending aorta under sodium thiopental anesthesia (50 mg/kg, intraperitoneally, Cristália, Brazil), for biochemical parameters assessment. Glucose levels and the lipid profile were assessed spectrophotometrically using colorimetric method (Celm, Brazil). Insulin was determined by radioimmunoassay (Linco, USA). The Homeostasis Model Assessment (HOMA-IR), an index of insulin resistance, was calculated from glucose and insulin levels, using the equation: fasting insulin (µIU/mL) × fasting glucose (mmol/L)/22.5 [24]. Lee's obesity index was calculated as follows: body weight1/3(g)/nasal-anal length (cm)x100. The white adipose tissue (epididymal and retroperitoneal) as well as gastrocnemius and long digital extensor muscles were weighted.

The blood pressure (BP) was measured in unanesthetized animals by indirect tail-cuff method (PowerLab 4/S, ADInstruments, Australia). Rats were maintained at 37°C for 10 min, and then three consecutive stable BP measurements were averaged.

### Plasma Concentrations of Angiotensin Metabolites by HPLC

Angiotensin peptide measurements were performed as described in detail previously [25]–[27]. A cocktail of protease inhibitors containing 1 mmol/l p-hydroxy-mercury benzoate, 30 mmol/l o-phenanthroline, 1 mmol/l PMSF and 1 mmol/l pepstatin A (140 µl per 1 ml of blood) was added to the blood samples; this mixture was then centrifuged at 1500 g at 4°C for 20 min and stored at −80°C until further analysis.

The plasma samples were concentrated in a C18 Sep-Pak column activated with sequential washes with methanol (5 ml), tetrahydrofuran (5 ml), hexane (5 ml), methanol (5 ml) and water (10 ml). The peptides were eluted with an ethanol/acetic acid/water (45∶2∶3) mix. The elutions were then freeze-dried and resuspended in 500 µl of mobile phase A [5% acetronitrile (50 ml) in 0.1% orthophosphoric acid (1 ml)]. The peptide was separated on a reverse-phase column [Aquapore ODS 300 (250 mm×4.6 mm)] using a gradient of 5–35% of mobile phase B (95% acetonitrile in 0.1% H3PO4) with a flow of 1.5 ml/min for 40 min in a Milton Roy System (containing two constaMetric 3000 pumps, a UV detector spectroMonitor 3100, a programmer GM 4000 and a mixer). Synthetic standards were used and peptide detection was carried out at 214 nm. The results are expressed in ng/ml.

### Vascular Function Studies

Force development in response to a specific experimental protocol was evaluated in mesenteric arteries from both groups as previously described [28]. The mesenteric vascular bed was removed and placed in modified Krebs-Henseleit solution of the following composition (in mM): 130 NaCl, 14.9 NaHCO_3_, 4.7 KCl, 1.18 KH_2_PO_4_, 1.17 MgSO_4_·7H_2_O, 5.5 glucose, 1.56 CaCl_2_·2H_2_O, and 0.026 EDTA. Segments (2 mm in length) of the mesenteric arteries were mounted on 40- µm wires in a small vessel myograph for isometric tension recording. The vessels were allowed to equilibrate for about 30 min in modified Krebs-Henseleit solution, which was gassed with 5% CO_2_ in O_2_ to maintain a pH of 7.4. The relationship between resting wall tension and internal circumference was determined, and the internal circumference, L100, corresponding to a transmural pressure of 100 mmHg for a relaxed vessel *in situ*, was calculated. The vessels were set to the internal circumference L1, given by L1 = 0.9xL100. The effective internal lumen diameter was determined as L1 = L1/π, and was between 200 and 300 µm. After stabilization, arterial integrity was assessed by stimulation of vessels with 120 mM KCl. Endothelial function was assessed by testing the relaxant effect of acetylcholine (ACh, 1 µM) on vessels precontracted with phenylephrine (1 µM). The failure of ACh to elicit relaxation of mesenteric arteries (which were previously subjected to rubbing of the intimal surface with a human hair) was taken as proof of endothelium removal.

### Experimental Protocols

Non-cumulative concentration–response curves to Ang II were performed in different segments of mesenteric arteries. The curves were performed on a top of a submaximal tone (30 to 40% of the maximum response) induced by norepinephrine (NE) to avoid rapid receptor desensitization in the mesenteric arteries that would diminish contraction to Ang II [29]. To determine whether the alterations of Ang II-evoked responses were receptor specific, cumulative concentration–effect curves induced by the adrenergic agonist NE were also obtained. To investigate if the contractile responses were dependent on the intact endothelium, responses were also assessed in endothelium-denuded arteries. The contractile responses to Ang II and NE were normalized by expressing them as the percentage of contraction relative to contractions induced by KCl in a concentration that produces almost maximum contraction (120 mM).

The role of specific Ang II receptors in the Ang II-induced responses was assessed in the presence of the AT1 receptor antagonist losartan (incubated at the concentrations of 0.3 µM and 10 µM) or the AT2 receptor antagonist PD 123319 (1 µM). In order to investigate the contribution of NO in the vascular effects of Ang II, mesenteric arteries were pretreated with Nω-nitro-L-arginine methyl ester (L-NAME, 100 µM), a NO synthase inhibitor. To determine the involvement of MAPKs on Ang II-induced contraction, concentration-effect curves to Ang II and NE were performed either in the absence (control) or in the presence of PD98059 (1 µM), a specific MEK/ERK1/2 inhibitor, SB203580 (1 µM), a catalytic inhibitor for p38 MAPK, or SP600125 (1 µM), an inhibitor of JNK. To elucidate the contribution of reactive oxygen species (ROS) in the vascular effects of Ang II, mesenteric arteries were pretreated with apocynin (100 µM), a NADPH inhibitor. Each preparation was tested with a single inhibitor and tissues were incubated with these agents for 30 minutes prior to the concentration-response curves.

### Western Blotting

Mesenteric arteries from control and obese rats were stimulated with Ang II (0.1 µmol/L) or vehicle for 10 min in the absence or in the presence of the AT1 receptor antagonist losartan (10 µM), the AT2 receptor antagonist PD123319 (1 µM), or the ERK1/2 inhibitor PD98059 (1 µM).

After the incubation protocols, vessels were frozen in liquid nitrogen and proteins were extracted (50 µg) and separated by electrophoresis on 10% polyacrylamide gels and transferred to nitrocellulose membranes. Nonspecific binding sites were blocked with 5% skim milk in Tris-buffered saline solution with Tween (0.1%) for 1 hour at 24°C. Membranes were incubated with antibodies (at the indicated dilutions) overnight at 4°C. Antibodies were as follows: anti-p38MAPK (Thr180/Tyr182), anti-ERK1/2 (Thr202/Tyr204), anti-SAPK/JNK (Thr183/Tyr185), anti-AT1, anti-AT2, anti-eNOs (1∶500, Cell Signaling), anti-phospho eNOs (1∶1000, Cell Signaling), and anti-α-actin (1∶20000, Sigma). After incubation with secondary antibodies, signals were revealed by chemiluminescence, visualized by autoradiography and quantified densitometrically. Results were normalized to α-actin expression and expressed as units relative to the control.

### Data Analysis and Statistical Procedures

The contractile responses are expressed as percentage of the response to KCl. The individual cumulative concentration-response curves to NE were fitted into a curve by non-linear regression analysis. pEC50 (defined as the negative logarithm of the EC50 values) and maximal response were compared by t-tests or ANOVA, when appropriated. The non-cumulative concentration-response curves to Ang II were compared by two way ANOVA. The Prism software, version 5.0 (GraphPad Software Inc., San Diego, CA, USA) was used to perform the analysis of these parameters as well as to fit the sigmoidal curves. Data are presented as mean ± SEM. N represents the number of animals used. P values less than 0.05 were considered significant.

### Drugs

Sodium thiopental was purchased from Cristália LTDA (São Paulo, Brazil). Acetylcholine, angiotensin II, apocynin, losartan, PD98059, SB203580, SP600125, PD123319, monosodium glutamate, norepinephrine and Nω-nitro-L-arginine methyl ester were purchased from Sigma Chemical Co (St. Louis, MO).

## Results

### General Characteristics of Monosodium Glutamate-Induced Obese Rats

General and biochemical characteristics of control and obese rats are depicted in Table 1. Sixteen-week-old obese rats displayed higher Lee index, fat mass, serum triglycerides and low density lipoprotein (LDL) cholesterol. In addition, enhanced HOMA-IR index and hyperinsulinemia were found in obese rats. No difference in BP levels was found between the groups.

**Table 1:** General characteristics of sixteen-week-old control and obese rats.

Parameter	C	MSG (n = 10)
Body weight (g)	392.3±4.92	358.6±8.26 *
Naso-anal length (cm)	26.02±0.29	23.44±0.08 *
Lee index (x100)	28.57±0.17	30.50±0.25 *
Retroperitoneal WAT (g/100 g)	0.78±0.05	2.75±0.14 *
Periepididymal WAT (g/100 g)	0.66±0.073	2.41±0.16 *
Soleus muscle (g/100 g)	0.035±0.002	0.036±0.002
Extensor digitorum longus muscle (g/100 g)	0.033±0.002	0.031±0.002
Triacylglycerols (mg/dL)	68.50±3.29	122.80±6.48 *
Total colesterol (mg/dL)	65.20±2.85	67.00±3.33
HDL-colesterol (mg/dL)	15.44±0.84	16.44±1.55
LDL-colesterol (mg/dL)	35.1±2.44	45.5±4.2 *
VLDL-colesterol (mg/dL)	13.7±0.66	24.54±1.29 *
Glucose (mg/dL)	97.1±3.49	93.9±2.69
Insulin (ng/mL)	13.7±0.97	25.6±2.52 *
HOMA-IR index	3.31±0.29	6.21±0.80 *
HOMA-β index	47.46±3.63	92.86±9.19 *
Angiotensin I (pg/mL)	32.95±1.22	81.54±5.10 *
Angiotensin II (pg/mL)	17.19±2.48	48.70±1.57 *
Angiotensin-(1–7) (pg/mL)	87.14±8.08	55.03±5.4 *
Blood Pressure (mmHg)	113.9±1.85	112.8±2.86

WAT, white adipose tissue; HDL, high density lipoprotein; LDL, low density lipoprotein; VLDL, very low density lipoprotein; HOMA-IR, homeostasis model assessment-insulin resistance; Values are mean ± SEM. *P<0.05 vs. control. N = 7–10/group.

Plasma measurements of angiotensin metabolites revealed that obese rats had an increase in both Ang I and Ang II concentrations when compared with control rats. Plasma levels of Ang 1–7 were significantly lower in obese rats when compared with control rats.

### Vascular Responses to Angiotensin II and Norepinephrine

The internal diameter of resistance mesenteric arteries (obese  = 236.7±4.4, n = 34; control  = 239.0±4.4, n = 39) as well as the contractile response to KCl (force in mN, obese  = 10.6±0.4; control  = 10.8±0.6) did not differ between obese and control rats.

**Figure 1:**
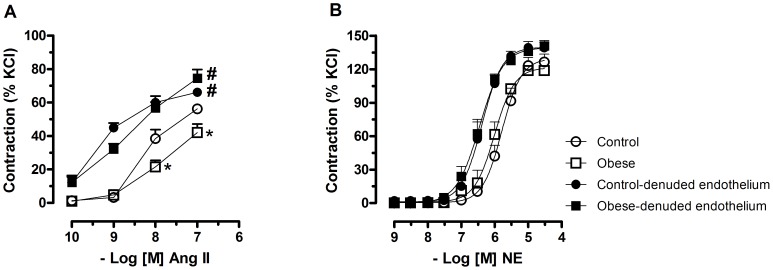
Effect of obesity on contraction of mesenteric arteries to angiotensin II and norepinephrine. **A-** Non-cumulative concentration–response curves to angiotensin II (ANG II) obtained in different segments of endothelium intact and endothelium denuded mesenteric arteries from control and monosodium glutamate (MSG)-induced obese rats. The curves were performed on a top of a submaximal tone (30 to 40% of the maximum response) induced by norepinephrine (NE). **B**- Cumulative concentration–response curves to NE in endothelium intact and endothelium denuded mesenteric arteries from control and monosodium glutamate-induced obese rats. Each point represents the mean ± SEM. *, P<0.05 vs. Control; ^#^, P<0.05 vs. respective group in the absence of endothelium. N = 5–6/group.

Ang II induced concentration-dependent contraction in mesenteric arteries from both obese and control rats. However, in preparations with intact endothelium from obese rats this response was impaired in comparison with the respective preparation of control rats. After endothelium removal, the response to Ang II was similar in preparations from obese and control rats (Figure 1A).

To determine whether the decreased vasoconstriction in obese rats was specific to Ang II, vascular reactivity to an alternative vasoconstrictor, NE, was examined. There were no differences in the contractile responses to NE between control rats and obese in preparations with or without endothelium (Figure 1B). Considering this, the studies were carried out with Ang II in endothelium-intact mesenteric arteries.

### Role of AT1 and AT2 Receptors on Angiotensin II–Induced Contraction

**Figure 2:**
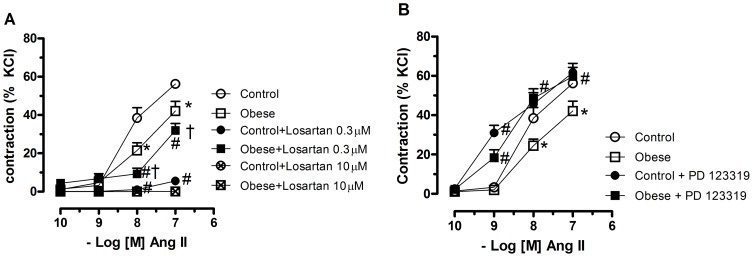
Contribution of angiotensin II receptors activation to the vascular effects of angiotensin. Mesenteric arteries with intact endothelium from control and monosodium glutamate-induced obese rats were pretreated with the AT1 receptor antagonist losartan (0.3 and 10 µM) (**A**) or the AT2 receptor antagonist PD 123319 (1 µM) (**B**), for 30 min and non-cumulative concentration–response curves to angiotensin II (ANG II) were obtained. Each point represents the mean ± SEM. *, P<0.05 vs. Control. ^#^, P<0.05 vs. respective group in the absence of blockade. ^†^, P<0.05 vs. Control + losartan. N = 5–6/group.

To determine the contribution of the AT1 receptor activation on Ang II effects in obese and control rats, vessels were pre-exposed to the selective AT1 antagonist losartan (0.3 µM). Incubation with losartan slightly reduced Ang II-induced contractile response in obese rats whereas it almost abolished this response in control rats (Figure 2A). Higher concentrations of the AT1 antagonist (10 µM) abolished the responses to Ang II in both groups.

We then examined whether AT2 receptor-mediated vasodilatation caused the impaired contraction to Ang II in mesenteric arteries from obese rats using the AT2 receptor antagonist PD 123319. Pretreatment with this antagonist augmented the responses to Ang II in preparations from both control and obese rats. However, force development in response to this peptide seems to be more dependent on AT2 receptors in preparations from obese rats since the effect of the antagonist in these rats was found to increase Ang II-elicited contraction at the three concentrations tested restoring the impaired contraction back to control levels, whereas in preparations from control rats only the response evoked by lower concentration of Ang II was increased by the antagonist (Figure 2B).

### Role of Nitric Oxide and Reactive Oxygen Species on Angiotensin II–Induced Contraction

**Figure 3:**
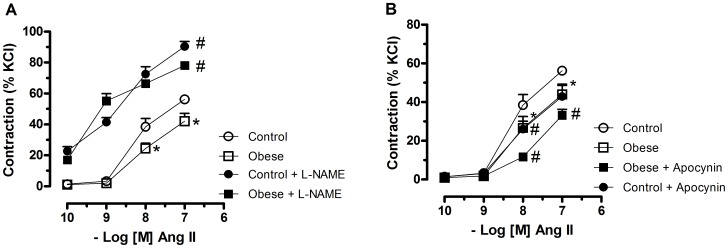
Contribution of nitric oxide and NADPH oxidase to the vascular effects of angiotensin. Mesenteric arteries with intact endothelium from control and monosodium glutamate-induced obese rats were pretreated with Nω-nitro-L-arginine methyl ester (L-NAME, 100 µM), a nitric oxide synthase inhibitor (**A**) or apocynin (100 µM), a NADPH inhibitor (**B**) for 30 min and non-cumulative concentration–response curves to angiotensin II (ANG II) were obtained. Each point represents the mean ± SEM. *, P<0.05 vs. Control. ^#^, P<0.05 vs. respective group in the absence of blockade. N =  6/group.

To investigate if the decreased response to Ang II in obese rats was due to increased NO release from the endothelium; concentration-response curves to Ang II were obtained in the presence of the NOS inhibitor L-NAME. In the presence of L-NAME, there was a similar increase in the efficacy of Ang II in vessels from both control and obese rats. In addition, similarly to the effects of endothelium removal, in the presence of L-NAME, no differences between segments of control and obese rats were observed, suggesting that the decreased response to Ang II in obese rats was dependent on NO release from the endothelium (Figure 3A).

Since Ang II activates NAD(P)H oxidases in endothelial and in vascular smooth muscle cells to produce ROS, which are involved in Ang II-induced vascular effects, the participation of these enzymes was assessed with apocynin, an inhibitor of NAD(P)H oxidase. Apocynin reduced the response to Ang II in mesenteric arteries from both obese and control rats. However, differently of the observed with L-NAME, the decreased response to Ang II in mesenteric arteries from obese rats was still observed after apocynin incubation (Figure 3B).

### Contribution of MAPKs on Angiotensin II-Induced Contraction

**Figure 4:**
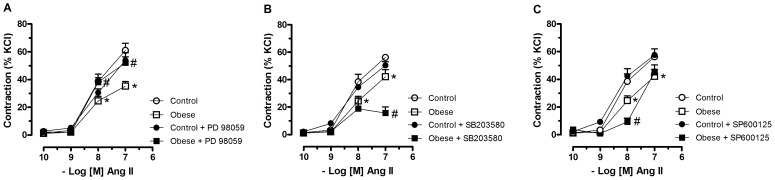
Involvement of MAPKs on angiotensin II-induced contraction. Mesenteric arteries with intact endothelium from control and monosodium glutamate-induced obese rats were pretreated with PD 98059 (1 µM), a specific MEK/ERK1/2 inhibitor (**A**), SB-203580 (1 µM), a catalytic inhibitor for p38 MAPK (**B**), or SP 600125 (1 µM), an inhibitor of JNK (**C**) for 30 min and non-cumulative concentration–response curves to angiotensin II (ANG II) were obtained. Each point represents the mean ± SEM. *, P<0.05 vs. Control. ^#^, P<0.05 vs. respective group in the absence of blockade. N =  6/group.

Incubation of mesenteric arteries with PD98059, an ERK1/2 inhibitor, corrected the reduced Ang II-induced response in obese without modifying the response to Ang II in control rats (Figure 4A).

Inhibition of JNK (Figure 4B) or p38 MAPKs (Figure 4C) did not modify the response to Ang II in control rats and reduced even more the contractile response to Ang II in mesenteric arteries from obese rats.

### Western Blot Analysis of Vascular AT1 and AT2 Receptors

**Figure 5:**
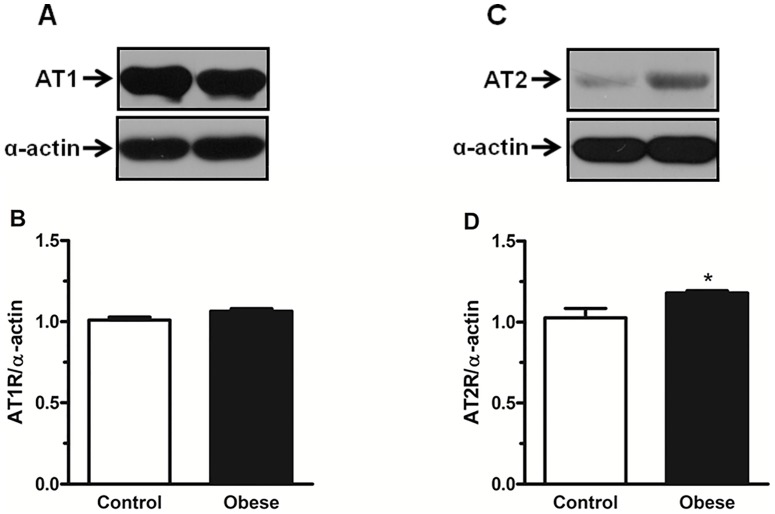
Effect of obesity on angiotensin II receptors protein expression in mesenteric arteries. Panels show densitometric analysis of the Western blots for AT1 and AT2 protein expression in endothelium intact mesenteric arteries from control and monosodium glutamate-induced obese rats. In **A** and **C**, Western blots for AT1 and AT2 receptors, respectively. Results were normalized to α-actin expression and expressed as units of change from the control. Data are expressed as mean ± SEM. *, P<0.05 vs. Control. N =  5/group.

Western blot analysis demonstrated that the protein expression of AT1 receptors in mesenteric arteries did not differ between from control and obese rats (Figure 5A). AT2 receptors were weakly expressed in mesenteric vessels control rats. However, in vessels from obese rats, protein expression of AT2 receptors was significantly increased (Figure 5B).

### Effects of Angiotensin II on ERK 1/2 Phosphorylation in Mesenteric Arteries

**Figure 6:**
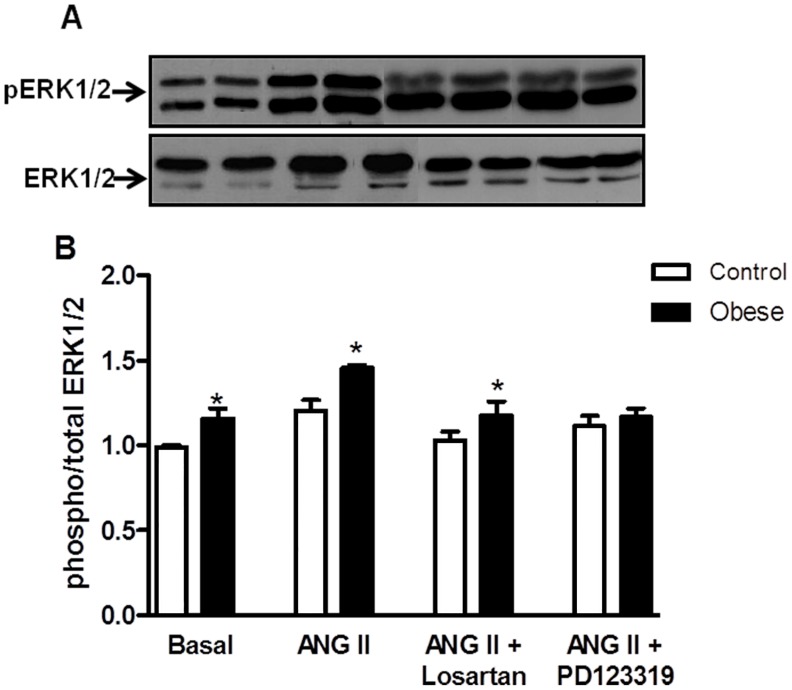
Effects of angiotensin II on ERK 1/2 phosphorylation in mesenteric arteries. Panels show densitometric analysis of the Western blots for ERK1/2 protein expression in endothelium intact mesenteric arteries from control and monosodium glutamate-induced obese rats. Vessels from both groups were stimulated with ANG II (0.1 µmol/L) or vehicle for 10 min in the absence or in the presence of the AT1 receptor antagonist losartan (0.3 µM, 30 min), the AT2 receptor antagonist PD 123319 (1 µM, 30 min) or the ERK1/2 inhibitor PD98059 (1 µM, 30 min) and the phosphorylation of ERK 1/2 was examined. Total protein levels are shown as loading controls. Data are expressed as mean ± SEM. *, P<0.05 vs. Control. N = 5–6/group.

As shown in figure 6, similar basal expression of ERK1/2 was found in vessels from control and obese rats. Ang II increased ERK1/2 phosphorylation in both groups and the magnitude of ERK1/2-induced phosphorylation was increased in mesenteric arteries from obese rats compared with control preparations.

To investigate the specific role of AT1 and AT2 receptors activation by Ang II on ERK1/2 phosphorylation, protein expression of ERK1/2 was examined in mesenteric arteries incubated with either losartan or PD123319 previously to the incubation with Ang II. Blockade of either AT1 or AT2 receptor attenuated ERK1/2 phosphorylation in mesenteric arteries from both control rats and obese rats.

### Effects of Angiotensin II on eNOS Phosphorylation in Mesenteric Arteries

**Figure 7:**
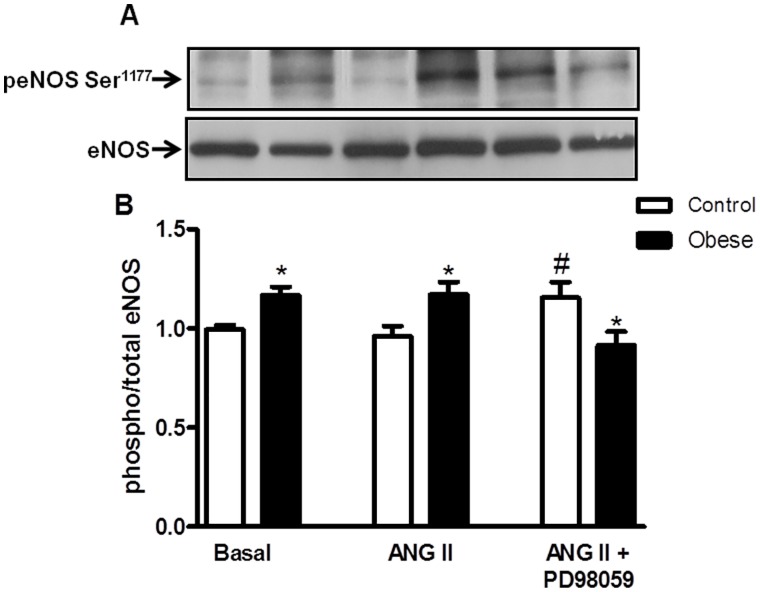
Effect of angiotensin II on eNOS phosphorylation in mesenteric arteries. Panels show densitometric analysis of the Western blots for eNOS protein expression in endothelium intact mesenteric arteries from control and monosodium glutamate-induced obese rats. Vessels from both groups were stimulated with ANG II (0.1 µmol/L) or vehicle for 10 min in the absence or in the presence of the ERK1/2 inhibitor PD98059 (1 µM, 30 min) and the phosphorylation of eNOS was examined. Total protein levels are shown as loading controls. Data are expressed as mean ± SEM. *, P<0.05 vs. Control, ^#^, P<0.05 vs. respective group in the presence of ANG II. N = 5–6/group.

Phosphorylation of eNOS was increased in vessels from obese rats compared to control rats in basal conditions. Stimulation with Ang II failed to induce eNOS phosphorylation in mesenteric arteries from both control and obese rats. Incubation of mesenteric arteries with the ERK1/2 inhibitor (PD98059) before Ang II stimulation increased eNOS phosphorylation in control rats, whereas it significantly decreased the phosphorylation of this enzyme in vessels from obese rats (Figure 7).

## Discussion

Cellular mechanisms and signaling pathways involved in the vascular dysfunction present in obesity are currently subjects of intensive investigation. In the present study, we highlighted the importance of AT2 receptors and MAPKs in the functional and molecular processes underlying the changes of vascular reactivity to Ang II in obesity. Major findings in the present study demonstrate that although MAPKs do not constitute the main mechanism involved in the vasoconstriction in control rats, these proteins are differentially regulated by Ang II in resistance mesenteric arteries from obese rats. While SAPK/JNK and p38MAPK pathways contribute to the maintenance of vasoconstriction to Ang II via AT1 receptors, activation of ERK1/2-eNOS pathway via AT2 receptors in the endothelium contributes to counteracting contraction and decrease the response to Ang II in obese rats.

In the present study, Ang II dose-dependently contracted small arteries from control and obese rats. However, responsiveness in vessels from obese rats was significantly lower than in control rats. This alteration appears to be receptor specific, because contraction induced by the adrenergic agonist NE was not affected in obese animals. Underlying mechanisms for the changes in Ang II–induced response in obese rats could be due to alterations in systemic levels of this peptide. In fact, enhanced activity of the RAS, represented by increased circulating angiotensinogen, renin, aldosterone, and angiotensin-converting enzyme activity has been reported in obesity [30]–[32]. Accordingly, an important finding in the present study is that circulating concentrations of Ang I and Ang II peptides were markedly increased in obese rats. Thus, the reduced contractile response to Ang II in these animals could represent a compensatory mechanism to counteract the increase in the synthesis and/or release of components of local and systemic RAS.

Since its discovery, the RAS has been considered an important component of the disturbances in the cardiovascular system. Several experimental data have demonstrated that Ang II is not the only biologically active component of the tissue and circulating RAS. Besides Ang II, several other angiotensin peptides have biological activity and are critically involved in the regulation of vascular function with important pathological implications. Among these is Ang-(1–7), a heptapeptide formed from Ang I or Ang II by either a carboxypeptidase called conversion enzyme or by tissue endopeptidases [33]. Ang-(1–7) exerts vascular relaxing actions mediated by activation of the proto-oncogene MAS product, stimulating similar pathways as AT2 activated by Ang II. The relaxing effect evoked by Ang-(1–7) results from both the potentiation of the dilating effects of bradykinin and the inhibition of angiotensin converting enzyme (ACE). Through these multiple pathways, Ang-(1–7) is considered an important component of a counter-regulatory axis within the RAS, comprised by ACE2/Ang-(1–7)/Mas receptor, which constitutes an intrinsic mechanism to induce vasoprotective actions by counter-regulating the ACE/Ang II/AT1R axis [34]. Indeed, the heptapeptide Ang-(1–7) has been described to have many beneficial effects in the vasculature that modulate the cardiovascular risk in obesity [35]–[37]. Accordingly, our findings that plasma levels of this peptide were decreased in obese rats point to a possible shift towards decreased activity of the ACE2/Ang-(1–7)/Mas receptor axis and increased ACE/Ang II/AT1R axis in mesenteric arteries as a causal mechanism for vascular dysfunction in obesity.

Ang II effects are thought to be regulated by the balance of AT1 and AT2 receptors expression. The AT1 receptor serves as a control point for regulating the ultimate effects of Ang II on its target tissues. Acutely, increased levels of Ang II may induce an increased activation of AT1 receptors; however, chronic exposure to high levels of Ang II evokes down regulation of AT1 receptors and/or upregulation of AT2 receptors [38], [39]. In this study, we demonstrated that obesity is accompanied by abnormal AT2 receptor upregulation in endothelium-intact mesenteric arteries, which most likely reduces Ang II-induced contraction even with no significant change in AT1 receptors expression. Consistent with our current findings, the upregulation of AT2 receptors and the role of this receptor mediating a reduced response to Ang II have been observed in other studies in the brush-border and basolateral membranes in obese Zucker rat [40], in the mesenteric arteries of young SHR [41], and in the thoracic aorta of SHR [42] and Goto-Kakizaki rat [14], a model of spontaneous normotensive type 2 diabetes. In fact, results from our study demonstrated that the selective AT2 receptor blocker PD123319 corrected the reduced response to Ang II in obese rats. Taking the above findings together, we speculate that in an early phase of obesity, AT2 receptor-mediated signaling pathways play a major role counteracting the effects of Ang II–mediated vasoconstriction as a consequence of the increased plasma levels of this peptide and the decreased levels of Ang-(1–7). Selective blockade of the AT1 subtype receptor with losartan antagonized the constrictor actions of Ang II in control group whereas it slightly decreased this response in obese rats. These data indicate that in control rats, Ang II–induced vasoconstriction in resistance mesenteric arteries is mediated exclusively via AT1 receptors, whereas in obese rats Ang II effects are associated with AT1- and PD123319-sensitive receptors, which may be AT2 receptors, as demonstrated by our findings.

It is well known that the endothelium plays an important role as a target of a variety of cardiovascular risk factors, including obesity and hypertension. Considering that Ang II is shown to induce the release of NO [43], [44], a role of endothelium and endothelium-derived NO on Ang II-induced vasoconstriction was determined in the isolated mesenteric arteries from control and obese rats. It is interesting to note that the endothelium removal or addition of L-NAME, an NOS inhibitor, not only increased Ang II-induced contraction per se in both control and obese rats, but made the contractile response to Ang II in mesenteric arteries from obese rats to be comparable to that of control rats. These findings lead us to postulate that the increased AT2 receptors expression is accompanied by eNOS upregulation in mesenteric arteries from obese rats, which depresses Ang II-induced contraction due to simultaneous stimulation of AT2 and eNOS. In fact, the pharmacological findings are supported by molecular data, where mesenteric arteries from obese rats displayed increased eNOS phosphorylation, probably contributing to attenuate the contractile response to Ang II in obese rats.

Interestingly, although basal expression of phosphorylated eNOS was augmented in vessels from obese rats, pretreatment with Ang II did not induce further increase in the activation of this enzyme, indicating that constitutively high enzymatic activity of eNOS appears to increase basal production of NO in obese rats, leading to attenuation of the vasoconstrictor effect of Ang II. In fact, this is supported by our findings that pretreatment with the ERK1/2 inhibitor restored the increased phosphorylation of eNOS and more importantly, that Ang II evokes ERK1/2 activation via both AT1 and AT2 receptors (as demonstrated by our western blotting studies, figure 5), indicating that the Ang II-induced augmented activation of ERK1/2 in vessels from obese leads to high enzymatic activity of eNOS.

We further probed the mechanisms leading to vascular activation of eNOS by Ang II. The observation that ERK1/2 inhibition with PD98059 before Ang II stimulation decreased eNOS phosphorylation in mesenteric arteries from obese rats indicates that ERK1/2-mediated eNOS phosphorylation contributes to decrease the response to Ang II in obese rats. Ang II has been shown to activate signaling cascades that activate MAPKs, including ERK1/2, JNK, and p38MAPK, which are implicated in VSMC differentiation, proliferation, migration, and fibrosis [4]–[6]. The ERK1/2 pathway is the best characterized of the MAPK pathways. Recent data implicates ERK1/2 in Ang II-mediated vascular smooth muscle contraction. Touyz et al. [45] showed that in vascular smooth muscle cells from human peripheral arteries, the ERK1/2 signaling cascade plays a role in Ca^2+^ pathways, which ultimately cause cell contraction. Enhanced activation of vascular ERK1/2 by Ang II has also been implicated in hypertension [46], [47]. Here we identify the ERK1/2 pathway as a putative mechanism counteracting contraction and decreasing the response to Ang II in obese rats. This is supported by our findings that incubation of mesenteric arteries with PD98059, an ERK 1/2 inhibitor, corrected the reduced response to Ang II in mesenteric arteries from obese rats without modifying the response to this peptide in control rats.

Additional evidence for a role of ERK1/2 mediating the reduced response to Ang II in obese rats was provided by western blotting experiments showing that Ang II-induced ERK1/2 phosphorylation is augmented in mesenteric arteries from obese rats. Interestingly, incubation with the AT2 receptor antagonist corrected the increased ERK1/2 phosphorylation in obese rats, indicating a dependence of AT2 receptors on Ang II-induced ERK 1/2 activation.

Besides activating ERK1/2, Ang II also stimulates JNK and p38MAPK, which are amongst the family of stress-induced kinases that influence cell survival, apoptosis, and differentiation. JNK and p38MAPK have also become known as important mediators of vascular contraction [48], [49]. However, no studies have so far attempted to investigate the role of JNK and p38 MAPK on Ang II-induced vascular contraction in mesenteric resistance arteries and their contribution to the changes of the vascular reactivity to Ang II in obesity. The findings of the present study provide evidence for a role of these MAPKs in vascular dysfunction in obesity. This is supported by the observations that inhibition of JNK and p38 MAPK further reduced the contractile response to Ang II in mesenteric arteries from obese rats but did not modify the Ang II-induced contraction in control rats. These results indicate that while SAPK/JNK and p38MAPK is not involved in contraction of mesenteric vessels from control rats, they contribute to the maintenance of vasoconstriction to Ang II in obese rats.

An increasing body of evidence has implicated oxidative stress in the vascular dysfunction present in insulin-resistant states, including diabetes, hypertension, and atherosclerosis. In this regard, the role of ROS production by NADPH oxidases in Ang II signaling, as well as a role for ROS in the development of different diseases in which Ang II is a central component has been extensively studied. Ang II activates NAD(P)H oxidases in endothelial cells and in vascular smooth muscle cells to produce ROS, such as superoxide and hydrogen peroxide (H_2_O_2_), which are involved in the pleiotrophic effects of Ang II [50]. The fact that inhibition of O_2_^−^ production by the NOX inhibitor apocynin, decreases vascular reactivity to Ang II in both control and obese rats, indicate that NOX-derived O_2_^−^ is importantly involved in the signaling pathways resulting in Ang II-induced vasoconstriction in mesenteric arteries. However, it is not involved in the changes of the vascular reactivity observed in obese rats.

## Conclusions

Our results demonstrate that MAPKs activity is differentially regulated by Ang II in resistance mesenteric arteries from obese rats. While SAPK/JNK and p38MAPK pathways contribute to the maintenance of vasoconstriction to Ang II, probably via AT1 receptors, activation of ERK1/2-eNOS pathway via AT2 receptors in the endothelium contributes to counteracting contraction and decrease the response to Ang II. These findings highlight the importance of local alterations in the RAS, rather than systemic effects, in mediating changes of the vascular function in obesity and also provide important information regarding the vasoconstrictor effects of Ang II that may be involved in vascular dysfunction associated with obesity.
